# Structural, Biochemical, and *In Vivo* Characterization of MtrR-Mediated Resistance to Innate Antimicrobials by the Human Pathogen *Neisseria gonorrhoeae*

**DOI:** 10.1128/JB.00401-19

**Published:** 2019-09-20

**Authors:** Grace A. Beggs, Yaramah M. Zalucki, Nicholas Gene Brown, Sheila Rastegari, Rebecca K. Phillips, Timothy Palzkill, William M. Shafer, Muthiah Kumaraswami, Richard G. Brennan

**Affiliations:** a Department of Biochemistry, Duke University School of Medicine, Durham, North Carolina, USA; b Department of Microbiology and Immunology, Emory University School of Medicine, Atlanta, Georgia, USA; c Department of Pharmacology and Chemical Biology, Baylor College of Medicine, Houston, Texas, USA; d Center for Molecular and Translational Human Infectious Diseases Research, Houston Methodist Hospital Research Institute, Houston, Texas, USA; e Department of Biochemistry and Molecular Biology, University of Texas, MD Anderson Cancer Center, Houston, Texas, USA; f Laboratories of Microbial Pathogenesis, VA Medical Research Service, Veterans Affairs Medical Center, Decatur, Georgia, USA; g Emory Antibiotic Resistance Center, Emory University School of Medicine, Atlanta, Georgia, USA; h Department of Biochemistry and Molecular Biology, Baylor College of Medicine, Houston, Texas, USA; i Department of Pathology and Genomic Medicine, Houston Methodist Hospital System, Houston, Texas, USA; Rutgers University-Robert Wood Johnson Medical School

**Keywords:** MtrR, *Neisseria gonorrhoeae*, bile salts, multidrug resistance, repression, structural biology, transcription

## Abstract

Neisseria gonorrhoeae responds to host-derived antimicrobials by inducing the expression of the *mtrCDE*-encoded multidrug efflux pump, which expels microbicides, such as bile salts, fatty acids, and multiple extrinsically administered drugs, from the cell. In the absence of these cytotoxins, the TetR family member MtrR represses the *mtrCDE* genes. Although antimicrobial-dependent derepression of *mtrCDE* is clear, the physiological inducers of MtrR are unknown. Here, we report the crystal structure of an induced form of MtrR. In the binding pocket of MtrR, we observed electron density that we hypothesized was *N*-cyclohexyl-3-aminopropanesulfonic acid (CAPS), a component of the crystallization reagent. Using the MtrR-CAPS structure as an inducer-bound template, we hypothesized that bile salts, which bear significant chemical resemblance to CAPS, are physiologically relevant inducers. Indeed, characterization of MtrR-chenodeoxycholate and MtrR-taurodeoxycholate interactions, both *in vitro* and *in vivo*, revealed that these bile salts, but not glyocholate or taurocholate, bind MtrR tightly and can act as bona fide inducers. Furthermore, two residues, W136 and R176, were shown to be important in binding chenodeoxycholate but not taurodeoxycholate, suggesting different binding modes of the bile salts. These data provide insight into a crucial mechanism utilized by the pathogen to overcome innate human defenses.

**IMPORTANCE** Neisseria gonorrhoeae causes a significant disease burden worldwide, and a meteoric rise in its multidrug resistance has reduced the efficacy of antibiotics previously or currently approved for therapy of gonorrheal infections. The multidrug efflux pump MtrCDE transports multiple drugs and host-derived antimicrobials from the bacterial cell and confers survival advantage on the pathogen within the host. Transcription of the pump is repressed by MtrR but relieved by the cytosolic influx of antimicrobials. Here, we describe the structure of induced MtrR and use this structure to identify bile salts as physiological inducers of MtrR. These findings provide a mechanistic basis for antimicrobial sensing and gonococcal protection by MtrR through the derepression of *mtrCDE* expression after exposure to intrinsic and clinically applied antimicrobials.

## Abstract (precis)

Neisseria gonorrhoeae causes a significant disease burden worldwide, and a meteoric rise in its multidrug resistance has reduced the efficacy of antibiotics previously or currently approved for therapy of gonorrheal infections. The multidrug efflux pump MtrCDE transports multiple drugs and host-derived antimicrobials from the bacterial cell and confers survival advantage on the pathogen within the host. Transcription of the pump is repressed by MtrR but relieved by the cytosolic influx of antimicrobials. Here, we describe the structure of induced MtrR and use this structure to identify bile salts as physiological inducers of MtrR. These findings provide a mechanistic basis for antimicrobial sensing and gonococcal protection by MtrR through the derepression of *mtrCDE* expression after exposure to intrinsic and clinically applied antimicrobials.

## INTRODUCTION

Neisseria gonorrhoeae, the causative agent of gonorrhea, is an exclusively human pathogen that causes more than 78 million infections worldwide annually (1, 2). Despite efforts to develop vaccines against gonorrhea, antibiotics currently remain the only choice for disease control (3). However, infection control by antibiotics is significantly challenged by the worldwide emergence of multidrug resistance in N. gonorrhoeae, which has prompted the Centers for Disease Control and Prevention to designate the gonococcus a “superbug.” Indeed, resistance to the last-line antibiotics cefixime and ceftriaxone, as well as azithromycin (used for dual therapy with ceftriaxone), has been reported in clinical isolates across the globe (2, 4, 5).

Among the various antibiotic resistance mechanisms employed by bacteria, efflux of diverse, structurally dissimilar antimicrobials by multidrug efflux pumps is implicated in intrinsic and acquired resistance (6–8). Among the characterized efflux pumps encoded by the gonococcal genome, *mtrCDE*, *farAB*, *macAB*, *norM*, and *mtrF* (9–13), the tripartite MtrCDE transporter is the best studied (14–19). MtrCDE belongs to the resistance-nodulation-division (RND) family of efflux pumps, which confers resistance against antimicrobial agents and a variety of clinically relevant antibiotics (15, 18–23). Indeed, genetic evidence suggests that various hydrophobic agents, including bile salts, fatty acids, and steroids, are substrates of MtrCDE (14, 17). Survival of gonococci that lack *mtrCDE* is significantly attenuated during experimental infection of the lower genital tract of female mice, suggesting that the action of the MtrCDE transporter is critical for bacterial fitness *in vivo* (24).

**FIG 1:**
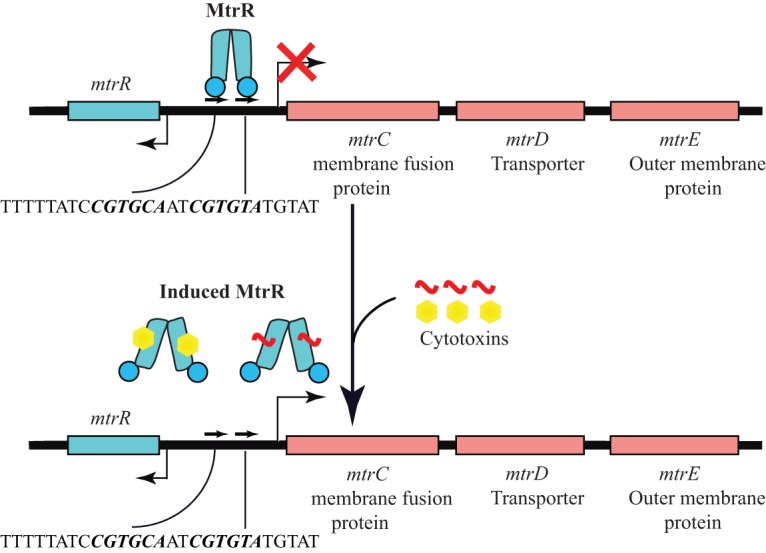
Transcription regulation of the *mtrCDE* and *mtrR* genes by MtrR. The colored boxes indicate the coding regions of the indicated genes. The bent arrows denote the transcription start sites of the respective genes. The red X signifies reduced transcription. The pseudo-direct repeat to which MtrR binds is shown schematically as pairs of arrows. The sequence of the 27-mer used in our DNA binding experiments is shown; the direct repeats in the sequence are shown in boldface italics. A cartoon representation of MtrR is shown in blue in its active and induced forms. Inducers of MtrR are labeled “cytotoxins” and shown in the MtrR-bound and free states.

The *mtrCDE* genes are organized in a three-gene operon, and their expression is repressed by the divergently transcribed transcription regulator MtrR (Fig. 1), which is a dimeric TetR family member with a predicted N-terminal helix-turn-helix (HTH) DNA binding motif and a C-terminal dimerization/inducer-binding domain (25, 26). Under physiological conditions, MtrR binds to a 27-bp direct repeat located upstream of the *mtrC* transcription start site in the intergenic region between the *mtrC* and *mtrR* genes and represses *mtrCDE* transcription, as well as that of its own gene (Fig. 1) (25, 27). As in other bacterial multidrug-binding regulators (28–30), MtrR likely responds to the influx of multiple cytotoxins into the cytoplasm by dissociating from the *mtrC* promoter and relieving repression of *mtrCDE* gene expression. In accordance with the critical role of the MtrR regulatory circuit in gonococcal multidrug resistance, drug-resistant clinical isolates frequently contain mutations either in the *mtrR* coding region or within the operator elements of the promoter, which leads to elevated expression of *mtrCDE* and correspondingly higher levels of antimicrobial resistance (23, 31). Further, gonococcal strains with an inactivated MtrR regulatory circuit also exhibited a competitive edge over wild-type (WT) strains in their growth in the lower genital tract of experimentally infected female mice (12, 24, 32). These observations suggest a role for MtrR-dependent gene regulation in gonococcal physiology beyond multidrug resistance. Consistent with this supposition, microarray analysis of wild-type and isogenic *mtrR*-inactivated gonococci revealed that, in addition to *mtrCDE*, MtrR also exerts significant influence, directly or indirectly, on the expression of approximately 67 genes, 45 of which are repressed and 22 activated (33).

Not surprisingly, several of the genes in the MtrR regulon are crucial for gonococcal response to oxidative, peroxide, and heat stress; evasion of host innate defense mechanisms; and host-pathogen interaction (33). Though the genetic basis of the significance of the MtrR regulatory network for gonococcal survival and pathogenesis is characterized, the mechanistic and structural bases of the signal-sensing mechanisms of MtrR are unknown. Indeed, relevant physiological inducers of MtrR and their direct interactions with MtrR have been neither characterized nor even identified.

In order to understand the mechanisms of cytotoxin sensing by MtrR, we carried out a number of structural, biochemical, and *in vivo* studies on this global regulator. Our structure of MtrR revealed electron density within the putative ligand-binding pocket that could be fit by a molecule of *N*-cyclohexyl-3-aminopropanesulfonic acid (CAPS), a buffer component of the crystallization solution. This allowed us to hypothesize that certain bile salts, which resemble CAPS and have been shown previously to reduce the viability of or elicit a combative response from N. gonorrhoeae, could bind MtrR and act as inducers (24). Our studies revealed that MtrR indeed utilizes these host-derived molecules, thereby likely providing N. gonorrhoeae the ability to overcome innate immunity, colonize the urogenital tract, and cause disease.

## RESULTS AND DISCUSSION

### Structure of MtrR.

The crystal structure of MtrR was determined to 2.40-Å resolution by multiple anomalous dispersion (MAD) methods using selenomethionine-derivatized MtrR (Semet-MtrR). Subsequently, the lower-resolution Semet-MtrR structure was used to determine the structure of the native protein by molecular replacement. The native structure was refined to 2.0-Å resolution with final *R*_work_ and *R*_free_ values of 20.4% and 24.7%, respectively. The asymmetric unit contains two MtrR dimers, with nearly all 210 residues of each protomer observed, with the exception of residues 1 through 7 in two subunits or 1 through 8 in the other subunits. This stretch is highly enriched in basic residues and likley plays a role in DNA binding. Other missing residues include loop residues 73 to 82, 76 to 81, and 76 to 83 in three of the four subunits that connect helices α4 and α5 and, in all four protomers, the carboxy-terminal residue 210.

**FIG 2:**
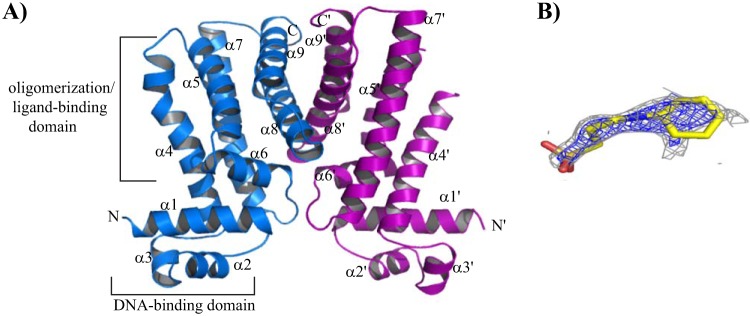
The induced structure of MtrR. (A) Cartoon of the induced structure of MtrR highlighting the potential binding site of CAPS, a hypothesized ligand necessary for crystallization. The individual subunits of MtrR are colored blue and purple, and the functional domains of one subunit are indicated and labeled. The secondary-structure elements of MtrR are labeled, and the primes indicate the structural elements from the second subunit. (B) CAPS fitted into electron density that is found in the putative inducer-binding pocket of all four independent subunits. The oxygen, nitrogen, and carbon atoms of the CAPS molecule are colored red, blue, and yellow, respectively. The composite Fo-Fc omit map contoured at 2.0 σ is shown in blue mesh, and the 2Fo-Fc map contoured at 1.0 σ is shown in gray mesh.

The subunits of each dimer are composed of nine α helices. Helix α1 is composed of residues 7 to 27; α2, 32 to 41; α3, 43 to 51; α4, 53 to 78; α5, 84 to 102; α6, 103 to 115; α7, 122 to 151; α8, 158 to 180; and α9, 185 to 204 (Fig. 2A). The root mean square deviation (RMSD) of the pairwise alignments of all corresponding Cα atoms of each subunit is 0.33 Å. The dimers are identical, as well; all corresponding Cα atoms can be superimposed, with an RMSD of 0.36 Å.

MtrR has two distinguishable functional domains: an N-terminal DNA binding domain with a helix-turn-helix (HTH) motif and a C-terminal dimerization/ligand-binding domain (Fig. 2A). A three-helix bundle in the N terminus contains the canonical HTH motif, with α3 being the “recognition helix” and α2 the “positioning helix” (Fig. 2A). The HTH motif of MtrR shares strong structural homology with the HTH motifs of the TetR family members Pseudomonas putida TtgR, Staphylococcus aureus QacR, and Campylobacter jejuni CmeR and is superimposed on the corresponding motifs of TtgR with an RMSD of 0.8 Å, QacR with an RMSD of 1.0 Å, and the bile salt-binding global transcription regulator CmeR with an RMSD of 2.3 Å. The poorer superposition of CmeR is the direct result of the disorder of its α3 in the absence of DNA. Helix α1 of MtrR lies nearly perpendicular to α3 and is preceded by the highly basic sequence MRKTK, which is poorly structured in each subunit and is posited to play a role in DNA binding, as is the N-terminal end of α1, which contains two positively charged residues. The presence of this large number of positively charged residues in MtrR (5 of the first 12 residues) is analogous to that of the respective 19- and 28-residue N-terminal extensions found in SimR and AmtR, two TetR family members that contain positively charged extensions critical for DNA binding (34, 35).

The six α-helices of the C terminus (α4 to α9) form the dimerization and ligand-binding domain (Fig. 2A). The dimer interface is formed primarily by the burial of hydrophobic residues in the middle of a four-helix bundle that is formed by α8 and α9 of each subunit. Additional contributions to dimerization come from the interactions between the loops connecting helices α6 and α7, as well as the loops between helices α1 and α2 of one subunit. Consequently, 1,780 Å^2^ of accessible surface area per protomer is buried in the formation of the MtrR dimer. The dimer interface is predominantly hydrophobic but is further bolstered by a number of salt bridges between the side chains of apposing subunits of the MtrR dimer, including R192-D158′, R176-D171′, and H204-D199′ (where the prime indicates the amino acids from the second subunit of the MtrR dimer) (see Fig. S1 in the supplemental material). The mode of dimerization in MtrR differs from those of QacR, CmeR, and TetR but resembles that of TtgR in that both MtrR and TtgR utilize the intervening loops between α6 and α7 and between α1 and α2, in addition to the C-terminal helices, in dimer formation (36).

### Location of the inducer-binding pocket.

The initial solvent-flattened electron density map revealed positive electron density in each of the four independent putative ligand-binding pockets of each MtrR protomer, suggesting a “ligand” was fortuitously captured during the purification or crystallization process. The density could be fit by CAPS, the biological buffer component of the MtrR crystallization reagent (see Fig. S2A in the supplemental material). No other crystallization or purification chemical component was structurally compatible with this mystery density. After refinement, a composite mFo-Fc omit map was generated, which also revealed distinct, albeit weak, density for CAPS (Fig. 2B and Fig. S2B). When the MtrR structure was analyzed by the ligand-binding site detection program Q-finder (37), the predicted sites clearly overlapped the CAPS binding site of each subunit. Analysis of the ligand-binding pocket of MtrR revealed an estimated volume of ∼1,000 Å^3^ (1,100 Å^3^ from Voidoo [Uppsala Software Factory] and 928 Å^3^ from Pocket Finder [omiX]). As observed in all other TetR family members, the multidrug-binding pocket of MtrR is located in the C-terminal domain with ligand-binding residues contributed from all C-terminal helices except α9. The lateral walls of this MtrR-ligand-binding site are formed by α4, α5, α7, and α8, which run in an antiparallel fashion, while the “floor” of the binding pocket is formed primarily by α6 (Fig. 2A).

A putative drug entry portal of MtrR resembles that of TtgR from P. putida rather than those of S. aureus QacR and C. jejuni CmeR, where the entry point of the ligands of the last two proteins is proposed to be located at the dimer interface (29). In contrast, the point of entry in MtrR and TtgR is situated on the lateral surface of the molecule between helices α4 and α7, away from the dimer interface (see Fig. S3 in the supplemental material) (36). It should be noted that the composite Fo-Fc omit map of the CAPS ligand does not completely cover the hydrophobic cyclohexane ring or sulfonic group of CAPS (Fig. 2B). Such weak or incomplete electron density of ligands bound to other multidrug-binding transcription factors has been observed. One such example is seen in the structure of QacR bound to two bivalent cationic compounds, which bind QacR with low micromolar affinities (38). In this more extreme case, QacR clearly assumed an induced conformation, but only noncontinuous electron density was scattered throughout the multidrug-binding site for each compound. Indeed, each ligand could be modeled into the pocket in multiple poses without resulting in steric clash. Thus, it is not surprising that a low-affinity “ligand” such as CAPS would also not display strong density throughout its entirety, especially given that the compound is not a physiologically relevant inducer and the inherent conformational flexibility of the cyclohexane ring and the rotatable sulfonate head group. While a CAPS molecule could fit the density within the putative binding pocket, we were not able to refine our structural model of CAPS. Indeed, removing CAPS from the pocket dropped the *R*_free_ by 2% and significantly improved the MolProbity clash score. Thus, our final structural model of MtrR does not contain CAPS.

Despite our inability to refine this mystery electron density with CAPS, its location provided insight into the chemical makeup of the putative MtrR binding pocket. This cavity is primarily composed of nonpolar residues and is populated with more than 20 hydrophobic or aromatic amino acid residues. The binding pocket also has 6 positively charged and two negatively charged residues. Fitting a CAPS molecule into each of the four subunits of MtrR in the asymmetric unit revealed that each CAPS molecule could occupy the same binding pocket and be coordinated by a similar set of MtrR residues. For example, in one MtrR dimer, the nonpolar cyclohexane ring of each CAPS molecule is most likely sandwiched between the aromatic side chains of residues F95 and F96 of α5 and W136 of α7 on one side of the wall and may interact with the side chains of residues L92 of α5 and L170 of α8 on the other side. The sulfonic group of CAPS appears to interact with side chains of residues from the same subunit, as well as from the other subunit, including D171 and R176′ (where the prime indicates the other protomer). The charge-neutralizing interaction between residue R176′ and CAPS is buttressed by its interactions with the side chain of residue D171. All the potential contact residues are shown in Fig. S4 in the supplemental material.

### Functional consequences of MtrR-CAPS interaction.

**FIG 3:**
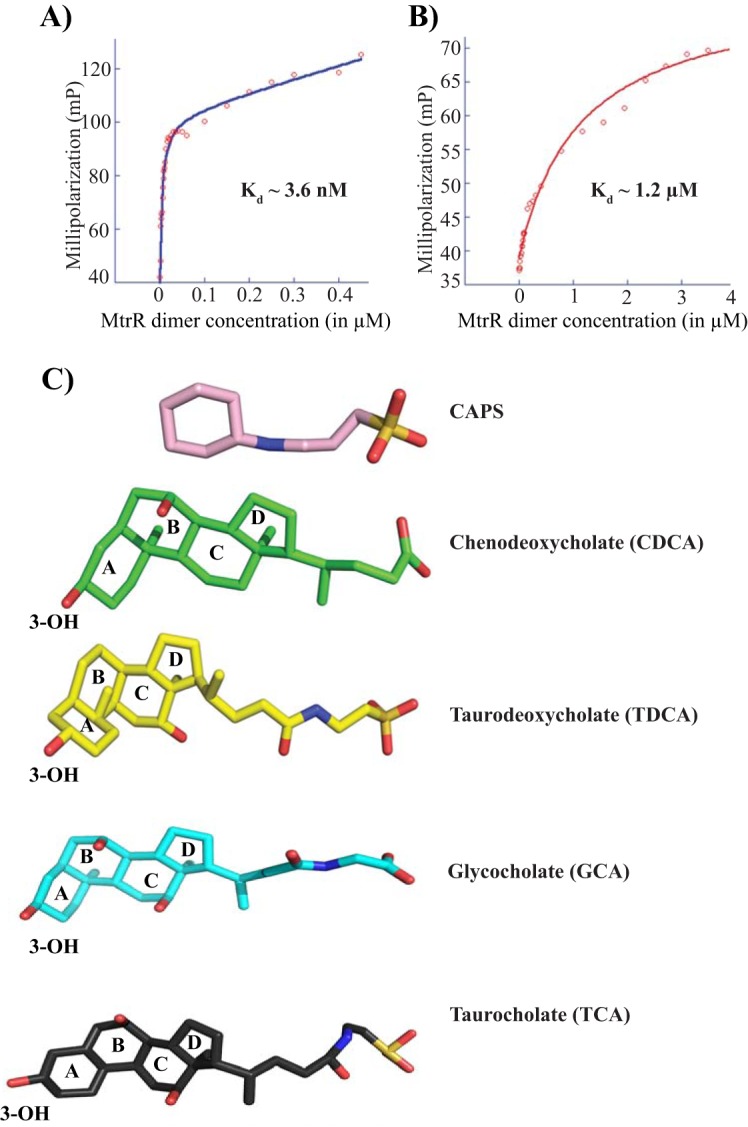
CAPS interferes with MtrR-DNA binding. The dissociation constants were determined using a fluorescence polarization-based assay for MtrR binding to a fluoresceinated oligoduplex containing an MtrR binding site in the absence (A) and presence (B) of 10 mM CAPS. (C) Chemical structures of the buffer CAPS and the bile salts CDCA, TDCA, GCA, and TCA. The ligands are presented as color-coded sticks. The four rings of the bile salts are labeled. Note the presence of at least one ring followed by a single-bonded chemical chain ending with a negatively charged group.

Given that each CAPS molecule appears to bind the proposed multidrug-binding pocket of MtrR, we tested the ability of CAPS to function as an inducer, albeit a nonphysiological inducer, by a fluorescence polarization (FP)-based DNA binding assay. Using a 5′-fluoresceinated 27-bp oligodeoxynucleotide duplex containing the MtrR binding site from the *mtrC* promoter, we measured the affinity of MtrR for its cognate DNA in the presence and absence of 10 mM CAPS. The crystallization condition of MtrR contains 100 mM CAPS buffer at basic pH (pK_a_, ∼10.5). Thus, to rule out any effect of alkaline pH on MtrR-DNA interactions, we buffered the CAPS used in the FP assay to pH 8.0 before testing its effect on DNA binding. This also resulted in the charge neutralization of the CAPS via protonation of its amino group. The data revealed that the affinity of MtrR for the *mtrC* operator sequence was reduced >300-fold (from a *K_d_* [equilbrium dissociation constant] of ∼3.6 nM to a *K_d_* of ∼1.2 μM) in the presence of 10 mM CAPS (Fig. 3A and B). These observations suggest that CAPS mimics the physiological inducers of MtrR and support our contention that the structure of MtrR in the presence of CAPS represents an induced state. In further accordance with this hypothesis, the structure of MtrR is incompatible with B-DNA binding, as the center-to-center distance between the recognition helices [L47(Cα) to L47′(Cα)] of MtrR is ∼45 Å, too far apart to interact with adjacent major grooves of B-DNA (see Fig. S5 in the supplemental material). Collectively, these data indicate that we have crystallized an induced form of MtrR and that CAPS may be chemically similar to physiologically relevant inducers of MtrR.

### MtrR directly interacts with bile salts, which interfere with MtrR-DNA binding.

Using the combination of the chemical structure of CAPS as a template and the relative abundance of substrates in the urogenital tract of females and males and rectum of both that are often infected by gonococci, attempts were made to identify the physiological inducers of MtrR. We observed that the chemical structures of bile salts display an intriguing resemblance to the chemical structure of CAPS (Fig. 3C). Each of the compounds has a terminal negatively charged group connected by a flexible linker to a ring structure, with bile salts containing a bulkier four-ring sterol group in contrast to the single-ring CAPS. Given the structural and chemical resemblances between CAPS and bile salts and the genetic evidence that the MtrCDE efflux pump recognizes bile salts as substrates (16), we postulated that one or more bile salts might be physiological inducers of MtrR.

**FIG 4:**
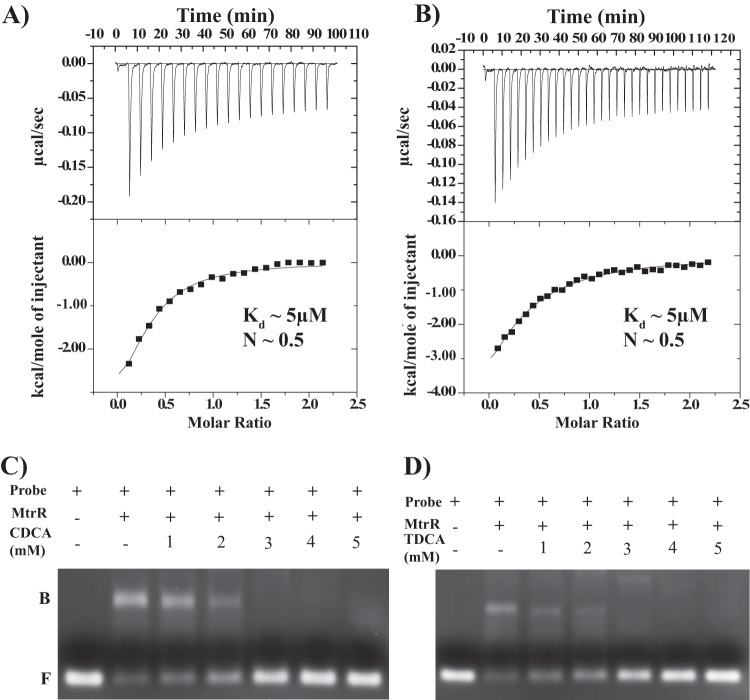
MtrR binding to and induction by CDCA and TDCA. (A and B) Isothermal titration calorimetry thermograms and resulting binding isotherms for the interactions between MtrR and CDCA (A) and MtrR and TDCA (B). (C and D) Electrophoretic mobility shift assays of the MtrR-DNA complex in the presence of CDCA (C) and TDCA (D). Preformed MtrR-*mtrCDE* promoter complexes were titrated against increasing concentrations of CDCA and TDCA. Each reaction was resolved on a 2% agarose gel and analyzed by staining with ethidium bromide. The positions of free probe (F) and MtrR-bound probe (B) are labeled.

To investigate whether MtrR directly interacts with selected bile salts, we carried out isothermal titration calorimetry (ITC) assays. The data revealed that MtrR binds directly to chenodeoxycholate (CDCA) and taurodeoxycholate (TDCA), each with a *K_d_* of ∼5 μM and each with stoichiometry of one molecule of bile salt per MtrR dimer (Fig. 3C and 4A and B; see Table S1 in the supplemental material). However, no measurable binding of glycocholate (GCA) to MtrR was detected by ITC (see Fig. S6 in the supplemental material). To test the hypothesis that these bile salts directly bind to MtrR and potentially derepress gene expression by interfering with the DNA binding activity of MtrR, MtrR-*mtrC* promoter interactions were tested in the presence and absence of different concentrations of several bile salts. Using an electrophoretic mobility shift assay (EMSA), three different bile salts, CDCA, TDCA, and taurocholate (TCA), were tested for the ability to dissociate MtrR from this cognate DNA binding site. Consistent with the ITC results, the addition of either chenodeoxycholate or taurodeoxycholate specifically disrupted the preformed MtrR-DNA complex at low millimolar concentrations (2 to 3 mM) (Fig. 4C and D), whereas the triple-hydroxylated bile salt taurocholate was much less efficient, failing to disrupt the MtrR-DNA complexes at a concentration of 10 mM (see Fig. S7 in the supplemental material). Glycocholate also failed to disrupt MtrR-DNA binding even at concentrations of 40 mM (data not shown). It is important to note that this is a competition assay that, due to its experimental design, requires the use of 3 μM DNA and 4 to 10 μM MtrR dimer. Consequently, this means that ∼99.9% of all DNA is MtrR bound. Because of the vastly different affinities of MtrR for cognate DNA (low nanomolar) and for specific bile salts in the absence of cognate DNA (low micromolar), concentrations of bile salt competitor significantly higher than its *K_d_* would be needed to “capture” MtrR, as it dissociates from its cognate DNA only very infrequently. This is consistent with the experimental data.

To quantify the effect of bile salts on the DNA binding affinities of MtrR, fluorescence polarization DNA binding assays were carried out in the presence of various concentrations of bile salts. Consistent with the results from the EMSA, the presence of chenodeoxycholate or taurodeoxycholate drastically affected MtrR-DNA interactions. At a 1 mM concentration of either bile salt, the affinity of MtrR for its cognate DNA was reduced from 8- to 28-fold, whereas in the presence of 5 mM either bile salt, no DNA binding by MtrR was detected (Table 1). However, the triple-hydroxylated bile salt, glycocholate, had no effect on MtrR-DNA interactions at 1 mM but resulted in a modest 6-fold reduction at 10 mM (Table 1). Taurocholate displayed poor ability to interfere with DNA binding, as the inclusion of 1 mM in the DNA binding buffer resulted in no change in affinity whereas 5 mM resulted in lowering of the binding affinity by only 7-fold (Table 1). Together, these results support the hypothesis that chenodeoxycholate and taurodeoxycholate are bona fide inducers of MtrR at physiologically relevant concentrations (20 mM) (39) and directly influence the DNA binding activity of MtrR. Further, it appears that the presence of an extra hydroxyl group on the bile salts glycocholate and taurocholate, as well as differences in their linkers, interferes with their ability to bind MtrR.

**TABLE 1:** MtrR-*mtrCDE* promoter binding constants in the presence or absence of selected bile salts

Bile salt	Concn (mM)	*K_d_* (nM)	Fold increase in *K_d_*^*a*^
No bile salt	0	18 ± 3	
Taurodeoxycholate	1	500 ± 21	28
	5	No binding	No binding
Chenodeoxycholate	1	150 ± 18	8.3
	5	No binding	No binding
Glycocholate	1	21 ± 0.8	1.2
	5	90 ± 7	5
	10	110 ± 16	6
Taurocholate	1	20 ± 6	1.1
	5	130 ± 10	7.2
	10	310 ± 21	17.2

a Fold change is the ratio of the *K_d_* in the absence of bile salt to the *K_d_*s in the presence of different concentrations of bile salt.

### Initial mapping of the binding pocket of chenodeoxycholate.

We hypothesized that the bile salts chenodeoxycholate and taurodeoxycholate occupied the binding pocket in which the CAPS electron density was observed. In our crystal structure, residues R176′ and W136 appear to be potentially important for H bonding and π stacking, respectively, with the ligand (see Fig. S4). Therefore, we generated and purified the single-site mutant MtrR proteins, MtrR(R176A), MtrR(R176E), and MtrR(W136L), to determine the importance of these residues for ligand binding (see the supplemental material) (40). First, we determined the capabilities of these single mutants to bind DNA with our FP-based assay. All the mutants were capable of binding the *mtrCDE* target sequence, with dissociation constants in the nanomolar range and all within 5-fold of the wild-type protein (see Fig. S7A). Next, we carried out EMSAs to determine if these mutants could be “induced,” i.e., dissociated from the *mtrCDE* operator DNA, by chenodeoxycholate and taurodeoxycholate; taurocholate was used as a noninducer control (see Fig. S7B). At higher concentrations (5 to 10 mM), taurodeoxycholate induced all three mutants. Similarly, chenodeoxycholate induced the MtrR(W136L) protein at concentrations of 5 to 10 mM. However, chenodeoxycholate did not induce either MtrR(R176A) or MtrR(R176E) to the same degree as the wild-type protein.

**FIG 5:**
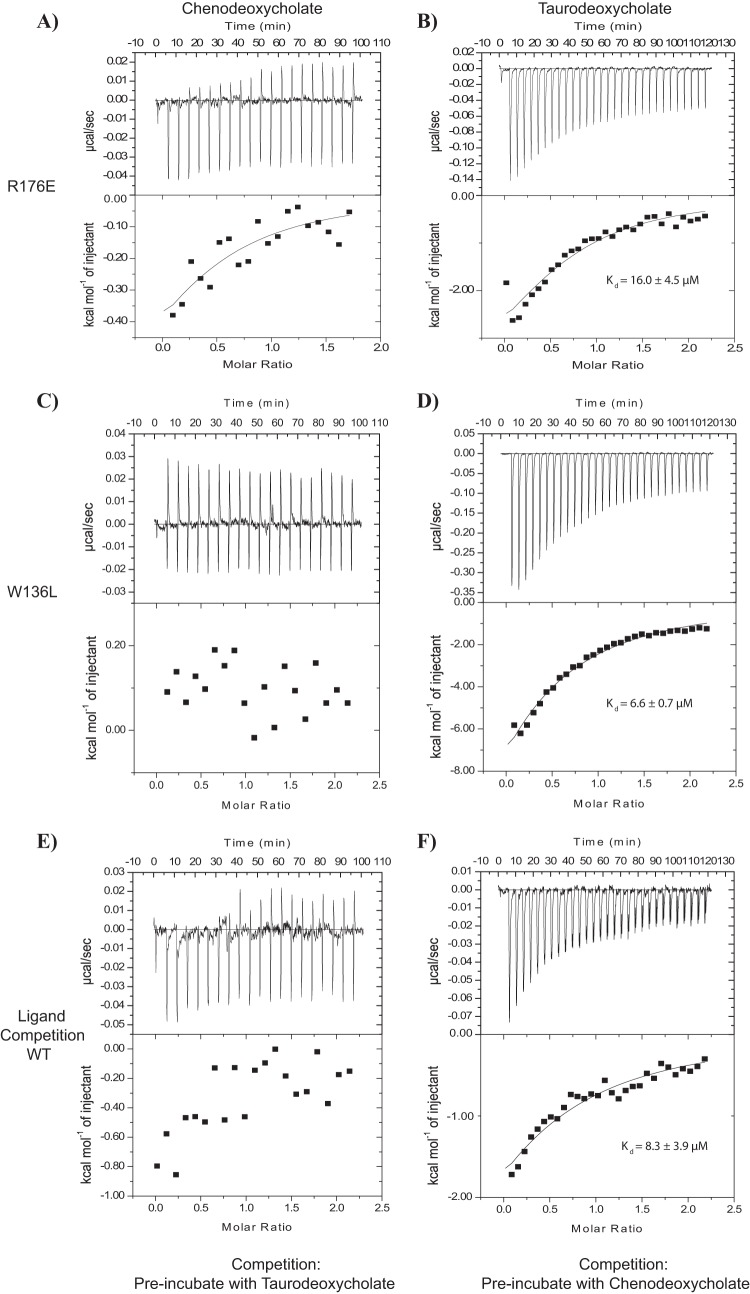
ITC experiments revealed two MtrR residues involved in binding chenodeoxycholate. (A to D) Isothermal titration calorimetry thermograms and resulting binding isotherms for binding reactions between MtrR(R176E) and chenodeoxycholate (A) and taurodeoxycholate (B), as well as between MtrR(W136L) and chenodeoxycholate (C) and taurodeoxycholate (D). (E and F) Isothermal titration calorimetry thermograms and resulting binding isotherms for ligand competition assays. (E) WT MtrR (20 μM) was incubated with 20 μM taurodeoxycholate overnight at 4°C; chenodeoxycholate was titrated into MtrR-TDCA. (F) WT MtrR (20 μM) was incubated with 20 μM chenodeoxycholate overnight at 4°C; taurodeoxycholate was titrated into MtrR-CDCA.

To quantitatively assess the ligand-binding capabilities of the mutant MtrR proteins, we performed ITC experiments with MtrR(R176E) and MtrR(W136L) (Fig. 5; see Table S1). MtrR(R176A) could not be purified in large enough quantities for ITC. Both mutant proteins had affinities of ∼5 μM for taurodeoxycholate, which is comparable to the *K_d_* of the wild-type protein (Fig. 5B and D). However, little to no binding was observed between the two mutants and chenodeoxycholate (Fig. 5A and C). It should be noted that MtrR(W136L) may still bind CDCA with a *K_d_* in the millimolar range. If this is the case, our ITC experiments would not be able to detect such binding because it is beyond the limits of detection of the instrument. In support of the supposition of a millimolar *K_d_*, when we used high concentrations of the bile salts (1 to 10 mM) in our EMSA, we observed that MtrR(W136L) was induced, i.e., dissociated from *mtrC* promoter DNA. Collectively, our gel shift and ITC assays indicated that R176′ and W136L are important for chenodeoxycholate binding and suggest that chenodeoxycholate occupies a binding site similar to the CAPS site (see Fig. S8 in the supplemental material).

To investigate these results further, we performed a ligand competition experiment in which MtrR was preincubated with saturating concentrations of either taurodeoxycholate or chenodeoxycholate, followed by titration of the second bile salt into the saturated protein-bile salt complex. Titration of chenodeoxycholate into MtrR presaturated with taurodeoxycholate showed minimal binding (Fig. 5E); however, binding was observed when taurodeoxycholate was titrated into MtrR saturated with chenodeoxycholate (Fig. 5F). This finding supports the idea that these ligands occupy different binding sites on MtrR and that the binding of taurodeoxycholate occludes, either directly or allosterically, the binding site of chenodeoxycholate. With our current data, we cannot determine the exact binding location of taurodeoxycholate. It is possible that taurodeoxycholate occupies a binding pocket similar to that of chenodeoxycholate but makes contacts with different residues; alternatively, it may occupy a binding site very different from that of chenodeoxycholate.

### Derepression of *mtrCDE* by bile salts.

To determine if sublethal concentrations of bile salts could relieve MtrR-mediated repression of *mtrC* (and, by inference, the *mtrCDE* operon), a translational fusion of the promoter region of *mtrC* to *lacZ* was used. This fusion system was made in the wild-type (FA19) background (41) and cloned into *mtrR* deletion mutant strain JF1. Using the *mtrCp-lacZ* fusion, the β-galactosidase activity of the strains grown in the presence or absence of the bile salt chenodeoxycholate, taurodeoxycholate, or glycocholate was measured. The β-galactosidase levels were determined from strains grown overnight on GC agar supplemented with the bile salt at concentrations 4-fold below their specific MICs to ensure that the concentration of each bile salt was in the same biological range (the bile salt concentrations are listed in Table 2).

**TABLE 2:** Fold increase of *mtrCp-lacZ* activity when strains were grown on sublethal concentrations of bile salts versus normal agar

Strain	Fold increase of activity^*a*^		
	Chenodeoxycholate	Glycocholate	Taurodeoxycholate
FA19	5.09 (0.2)	1.43 (0.48)	2.89 (0.3)
JF1 (FA19 Δ*mtrR*)	1.37 (0.2)^*b*^	2.11 (0.96)	1.33 (0.3)^*b*^

a Concentrations (millimolar) of bile salts included in the growth medium are given in parentheses.

b Statistically significant change (*P* < 0.001) compared to FA19 change.

For two of the tested bile salts, chenodeoxycholate and taurodeoxycholate, the MtrR-dependent derepression of the *mtrC* promoter was clearly evident, and a statistically significant loss of *mtrC* derepression in the *ΔmtrR* strain was observed compared to the MtrR-positive parent strain, FA19 (Table 2). However, the triple-hydroxylated bile salt, glycocholate, failed to exhibit MtrR-dependent derepression of the *mtrC* promoter. These results suggest that gonococci grown on sublethal levels of these two bile salts induce *mtrC* expression only in WT strain FA19 with an intact *mtrR* gene, which supports the hypothesis that specific bile salts act as potent physiological inducers of MtrR and relieve the repression of *mtrC* expression in an MtrR-dependent manner.

### Conclusions.

In conclusion, while much of the research on efflux pumps like MtrCDE has been focused on their multidrug-binding and transport properties and resistance against extrinsically administered drugs, their physiological role to safeguard the organism from endogenous antimicrobial substances within the host, such as bile salts, is often overlooked. Recent data strongly suggest that the multidrug resistance phenotype conferred by the efflux pumps is an evolutionary by-product of their primary role as an enhancer of bacterial survival and pathogenicity by shielding the organism from the toxic effects of host antimicrobials. Efflux-mediated tolerance of host defense mechanisms and its role in colonization and virulence have been shown for various enteric pathogens and N. gonorrhoeae (28, 42–45). The colonization surface for N. gonorrhoeae includes both urogenital tract and extraurogenital sites (rectum, eye, and oropharyngeal mucosae). In the rectum, colonization by gonococci is challenged by bile salts, which are detergent-like molecules with antimicrobial properties (39) present at concentrations as high as 20 mM, and MtrR-negative or MtrR-inactive mutants are often isolated from this site (14). The presence of cytotoxic chemicals on the colonization surfaces used by gonococci or within phagocytes mandates that the organism acquire defense mechanisms (46, 47).

Gonococci employ the efflux pump MtrCDE to evade the first line of the host innate defense by conferring low-level resistance against host cytotoxins, such as bile salts. The findings of the current study provide the first visualization and further biochemical characterization of MtrR, the key N. gonorrhoeae transcription regulator of a crucial multidrug efflux pump. Furthermore, this study shows biochemically that MtrR senses endogenous toxins produced by human neisserial hosts. In this respect, the presence of natural MtrR binding chemicals, such as bile salts (as described here) or other compounds, could provide a transient increase in gonococcal resistance to MtrCDE efflux pump antimicrobial substrates. This hypothesis is consistent with previous findings that in a female-mouse model of lower genital tract infection loss of MtrR resulted in enhanced resistance of gonococci to antimicrobials (32). We hypothesize that in this model of infection, the presence of bile salts could dysregulate MtrR repression of *mtrCDE*, but the type and concentration of bile salts at this site during infection are unknown. Alternatively, other inhibitory MtrR binding compounds (e.g., cationic antimicrobial peptides) could similarly increase the resistance of wild-type gonococci to host antimicrobials or currently used antibiotics exported by MtrCDE.

## MATERIALS AND METHODS

### Protein overexpression and purification.

Protein overexpression was performed as described previously (25) with the following modifications of the protein purification protocol. Cell pellets were resuspended in 50 ml of buffer A (20 mM Tris HCl [pH 8.0], 200 mM NaCl, 10% glycerol, and 1 mM tris-2-carboxyethyl phosphine hydrochloride [TCEP]) and lysed by an M-110L microfluidizer (Microfluidics). The protein was purified from the clarified cell lysate by Ni-nitrilotriacetic acid (NTA) affinity chromatography to >95% homogeneity. The hexahistidine-tagged MtrR was cleaved by thrombin (GE Healthcare) digestion, and the cleaved MtrR was purified from tagged MtrR and the cleaved hexahistidine tag by Ni-NTA affinity chromatography. Further purification was performed by size exclusion chromatography (S200 column), and the purified protein was concentrated to ∼30 mg/ml using an Amicon YM-10 membrane filter. Semet-MtrR was overexpressed using the methionine-inhibitory pathway (48) and purified as described for native MtrR.

### Crystallization, data collection, and structure determination.

Crystallization of native MtrR and Semet-MtrR was carried out using hanging-drop vapor diffusion methods with a crystallization solution containing 1.6 M Na^+^/K^+^ phosphate, 0.2 M LiSO_4_, and 0.1 M CAPS, pH 10.5. Semet-MtrR and native crystals were flash frozen using 10% glycerol as a cryoprotectant and MAD and single-wavelength diffraction data for Semet-MtrR and native crystals, respectively, were collected under cryogenic conditions at the Advance Light Source (ALS) on beam line 8.3.1. X-ray intensity data were processed with MOSFLM (49, 50) and SCALA (51). The MAD data were collected and processed to 2.4-Å resolution, and the native data were collected and processed to 2.0-Å resolution. It should be noted that the native data from 2.4- to 2.0-Å resolution contained ice rings. The crystals assumed the space group C 1 2 1 (C2), with the following unit cell dimensions: *a* = 218.3 Å, *b* = 84.6 Å, *c* = 58.1 Å, and β = 103.90°. For the MAD data set, 12 of 16 selenium sites were located using SOLVE (52), and density modification was carried out using RESOLVE (53). Utilizing the model built from the MAD data set and Phaser (54), the native MtrR structure was solved to 2.0-Å resolution (the limiting resolution of the native data set). After iterative rounds of model building using COOT (55) and refinement and validation using Phenix (56–59), the final refined model had an *R*_free_ of 24.7% and an *R*_work_ of 20.4% and was visualized with PyMol (60). The final model contained residues 7 through 209 of MtrR, 6 phosphate ions, and 242 water molecules. Selected data collection and refinement statistics are summarized in Table 3.

**TABLE 3:** Selected crystallographic data and refinement statistics

Parameter	Value(s)^*b*^	
	Semet-MtrR	Native
Data collection and phasing		
Wavelength (λ) (Å)	0.9797, 0.9798, 1.02	0.9797
Resolution (Å)	50.0–2.40	50.0–2.00
Overall *R*_sym_^*a*^	0.091 (0.38), 0.070 (0.28), 0.070 (0.21)	0.097 (0.90)
Overall *I*/σ(*I*)	12 (2.8), 14.7 (3.9), 16.3 (4.9)	8.4 (2.0)
Total no. of reflections	134,162, 132,575, 133,159	143,663
No. of unique reflections	35,267, 35,190, 35,256	65,953
Completeness (%)	99.9 (99.9), 99.8 (99.8), 99.9 (99.9)	98.2 (98.2)
No. of selenium sites	12/16	
Overall figure of merit^*c*^	0.78	
Refinement statistics		
Resolution (Å)		50.0–2.00
*R*_work_/*R*_free_ (%)^*d*^		20.4/24.7
Overall CC^*e*^		0.835
(CC_work_/CC_free_ high resolution)		(0.62/0.56)
No. of protein atoms		6,009
B factors (Å^2^)		55.5
No. of phosphate ions		6
Solvent no.		242
RMSD		
Bond lengths (Å)		0.006
Bond angles (°)		0.742
B for bonded main-chain atoms (Å^2^)		3.61
Ramachandran analysis: most favored/additionally allowed (%)		99.1/0.9

a *R*_sym_ = ΣΣ|*I*_hkl_ − *I*_hkl(_*_j_*_)_|/Σ*I*_hkl_, where *I*_hkl(_*_j_*_)_ is the observed intensity and *I*_hkl_ is the final average intensity value.

b Values in parentheses are for the highest-resolution shell. When three values are listed in a single row, they are associated with the data set collected at the 0.9797 Å, 0.9798 Å, or 1.02 Å, respectively.

c Figure of merit = <|Σ*P*(α)*e*^ia^/Σ*P*(α)|>, where α is the phase and *P*(α) is the phase probability distribution.

d *R*_work_ = Σǁ*F*_obs_| − |*F*_calc_ǁ/Σ|*F*_obs_| and *R*_free_ = Σǁ*F*_obs_| − |*F*_calc_ǁ/Σ|*F*_obs_|, where all reflections belong to a test set of 5% randomly selected reflections and *F*_obs_ is observed structure factor.

e CC, correlation coefficient.

### Isothermal titration calorimetry.

Purified MtrR was dialyzed extensively against buffer containing 20 mM Tris [pH 8.0], 200 mM NaCl, 10% glycerol, and 1 mM TCEP overnight at 4°C. The stock solutions and the necessary dilutions of the bile salts were prepared using this dialysis buffer. Titrations with 20 μM MtrR in the cell and 250 μM ligand in the syringe were performed using a VP-ITC microcalorimeter (Microcal Inc.). All measurements were conducted at 25°C with a stirring speed of 200 rpm. After subtraction of germane blank data, the titration data were analyzed using the program ORIGIN 5.0.

### Electrophoretic mobility shift assay.

Complementary oligodeoxynucleotides containing the MtrR binding site from the *mtrC* promoter (top strand, 5′-TTTTTATCCGTGCAATCGTGTATGTAT-3′; an unusual pseudo-direct repeat is underlined) were annealed by heating an equimolar mixture of the top and bottom strands at 95°C for 5 min, followed by slow cooling to room temperature. Binding reactions were carried out in 20-μl volumes of binding buffer (20 mM Tris [pH 8.0], 200 mM NaCl, 10% glycerol, and 1 mM TCEP) containing 1 μg oligoduplex, 1 μg poly(dI-dC), and 4 to 10 μg MtrR. After a 20-minute incubation at room temperature, the preformed complex was incubated with or without various concentrations of selected bile salts at room temperature for an additional 10 min. The reaction mixtures were resolved on a 2% agarose gel by electrophoresis for 45 min at 100 V at room temperature in Tris-acetate-EDTA (TAE) buffer. The gels were stained with ethidium bromide and visualized and analyzed with an Alpha Innotech gel documentation instrument. The intensities of free DNA were measured with ImageJII (61, 62).

### Fluorescence polarization-based DNA binding assay.

Fluorescence polarization-based DNA binding experiments were performed with a Panvera Beacon 2000 fluorescence polarization system (Invitrogen) utilizing 5′-fluorescein-labeled DNA. Polarization (*P*) of such 5′-labeled DNA increases as a function of protein binding, and equilibrium dissociation constants are determined by curve fitting the values of millipolarization (*P* × 10^−3^) units against the protein concentration. To determine the effects of various ligands on MtrR-DNA binding, 1 nM 5′-fluoresceinated oligodeoxynucleotide duplexes containing the *mtrC* operator site in 1 ml binding buffer (20 mM Tris HCl [pH 8.0], 100 mM NaCl, and 2.5% glycerol) were titrated against increasing concentrations of purified MtrR in the presence or absence of the indicated concentrations of bile salt, and the resulting changes in polarization were measured. All the samples were excited at 490 nm, and their polarized emissions were measured at 530 nm. All the data were plotted using Kaleidagraph (Synergy Software) and Prism (GraphPad Software), and the resulting plots were fitted to the following equation: *P* = {(*P*_bound_ − *P*_free_)[protein]/(*K_D_* + [protein])} + *P*_free_, where *P* is the polarization measured at a given protein concentration, *P*_free_ is the initial polarization of the free ligand, *P*_bound_ is the maximum polarization of specifically bound ligand, *K_D_* is the equilibrium dissociation constant, and [protein] is the protein concentration. Nonlinear least-squares analysis was used to determine *P*_bound_, and *K_d_*. The reported binding constants are the average values from at least three independent experimental measurements.

### β-Galactosidase assay.

N. gonorrhoeae strains FA19 (wild type) and JF1 (FA19 Δ*mtrR*) containing a translational *mtrCp-lacZ* fusion with the *proAB* locus were used to assess the ability of bile salts to induce *mtrC* expression; the construction of these strains has been described previously (41). The MIC of bile salts for each strain used in the β-galactosidase assay was determined using a 2-fold agar dilution method (32). Gonococci were grown overnight on GC agar (Difco Laboratories) supplemented with a bile salt (glycocholate, chenodeoxycholate, or taurodeoxycholate) at concentrations that were 4-fold below the MIC. After overnight growth at 37°C under 3.8% (vol/vol) CO_2_, the bacteria were scraped from the agar plate, resuspended in 1 ml phosphate-buffered saline (pH 7.4), and lysed by freeze-thawing three times, after which the cell debris was pelleted by centrifugation. The lysate was then used to determine β-galactosidase levels as described previously (63). All experiments were performed in triplicate with at least three biological replicates conducted on different days.

### Accession number(s).

The coordinates and structure factors for the MtrR-CAPS complex structure have been deposited in the RCSB Protein Data Bank with the accession code 6OF0.

## Supplementary Material

Supplemental file 1
