# Supplementary material for: Structural, Biochemical, and In Vivo Characterization of MtrR-Mediated Resistance to Innate Antimicrobials by the Human Pathogen Neisseria gonorrhoeae
Source: J Bacteriol. 2019 Sep 20;201(20):e00401-19. doi: 10.1128/JB.00401-19 (PMC6755732; doi:10.1128/JB.00401-19)
Supplement: Supplemental file 1 [file JB.00401-19-s0001.pdf]

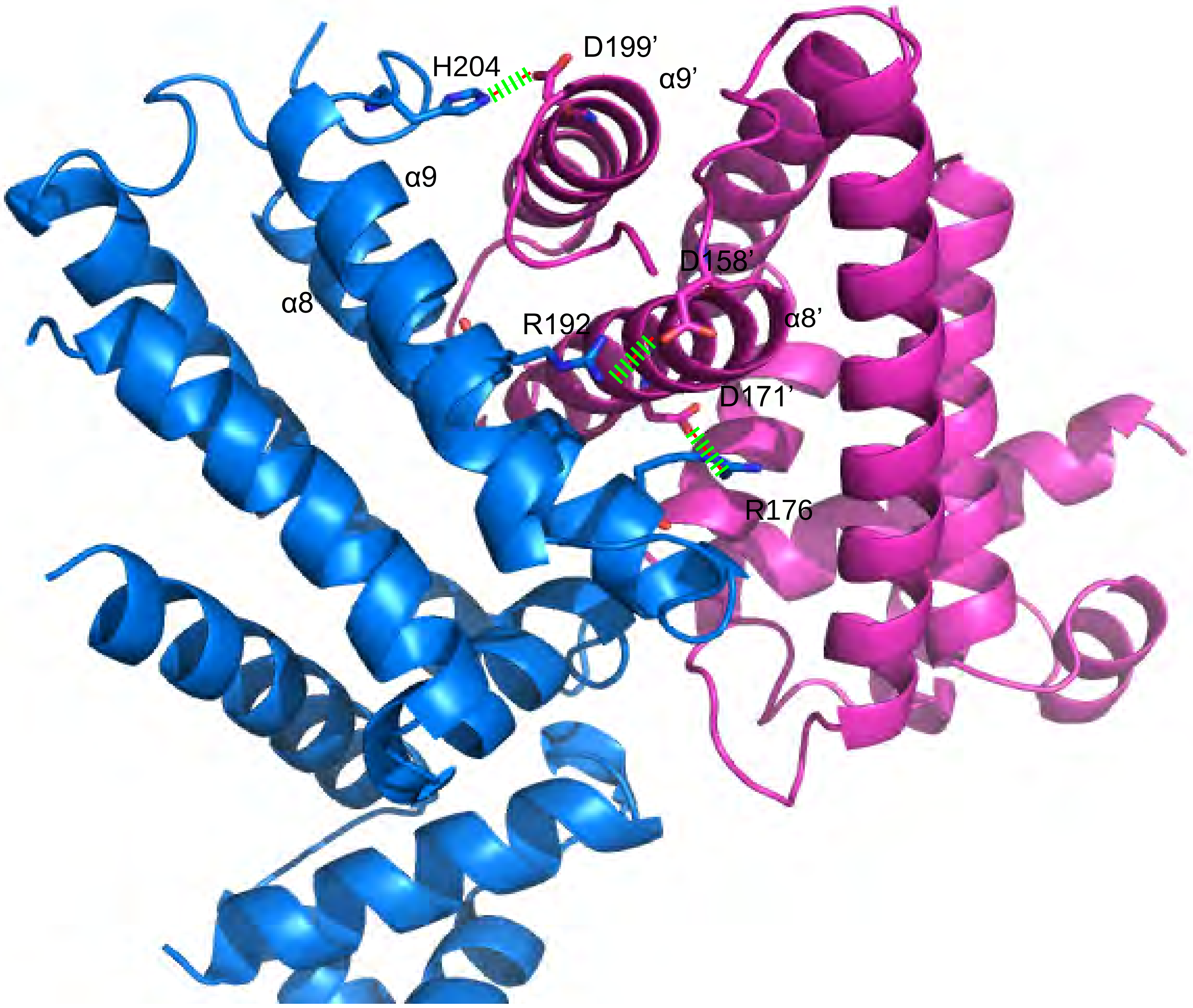

Supplemental Figure S1. Salt bridges of MtrR are shown by green dashes and the participating residues are labeled.

Subunits of MtrR are shown in blue and purple. The critical helices for dimerization (α8, α8’, α9, and α9’) are labelled.

A)

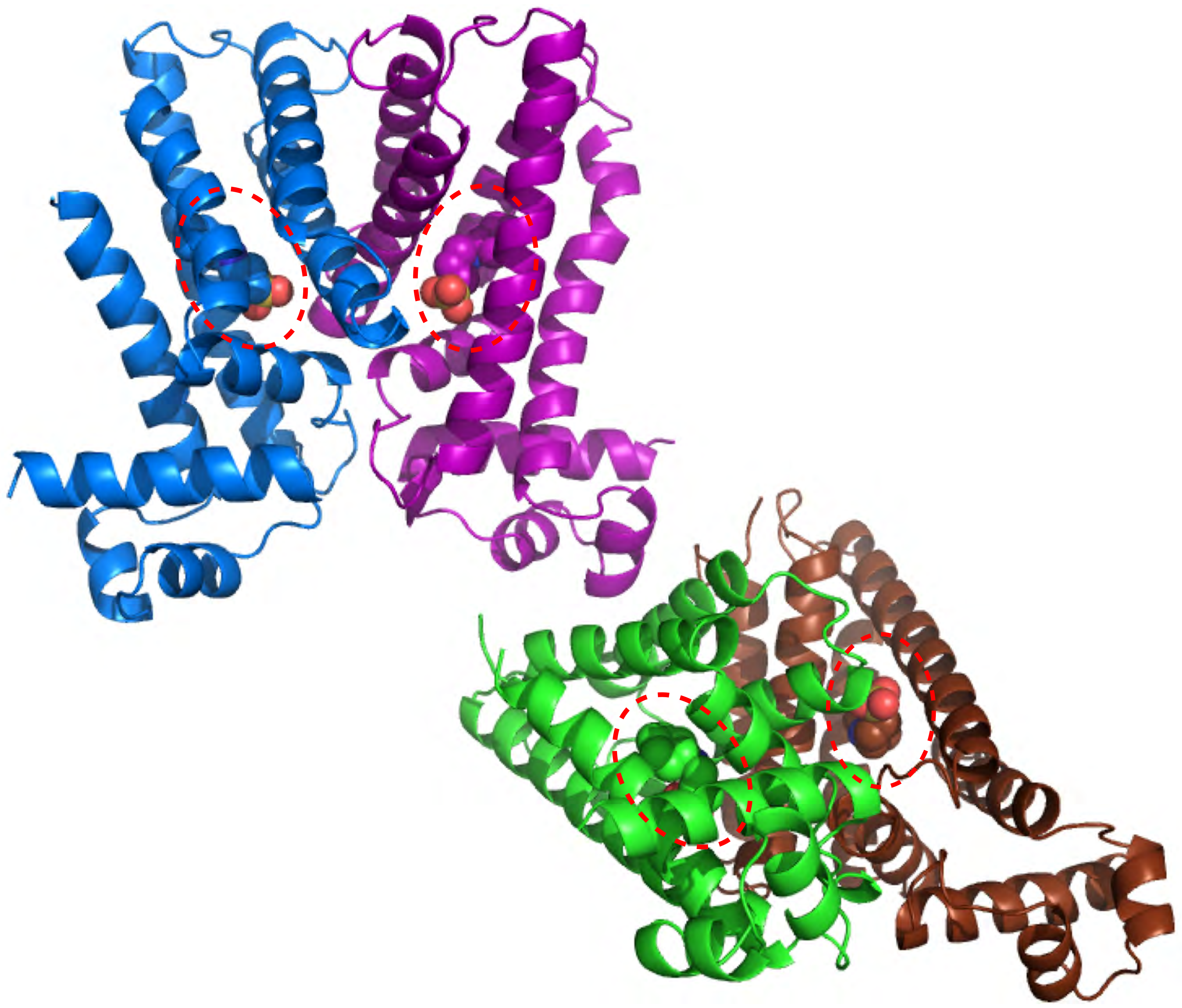

B)

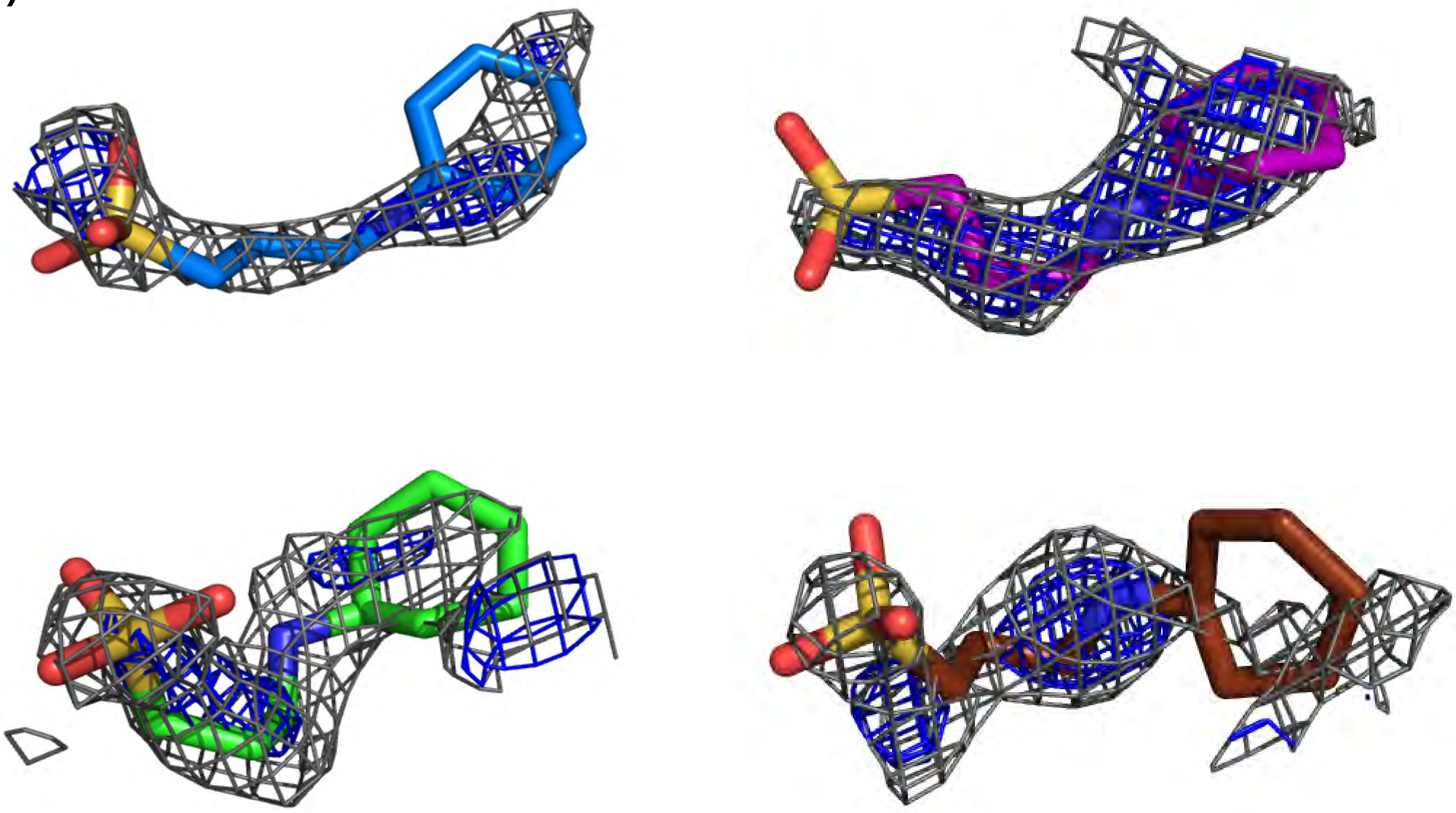

Supplemental Figure S2. CAPS molecules in asymmetric unit. **(A)** A cartoon of the four protomers of MtrR in the assymetric unit with each bound CAPS molecule displayed as spheres within the ligand binding pocket. Each CAPS molecule is colored to match the color of its corresponding MtrR subunit and is denoted by a red dashed circle. **(B)** Each CAPS molecule in the asymmetric unit is displayed with its electron density; the colors of the CAPS molecule correspond with the colors in (A). The composite Fo-Fc omit map contoured at 2.0σ is shown in blue mesh and the 2Fo-Fc map contoured at 1.0σ is shown in grey mesh.

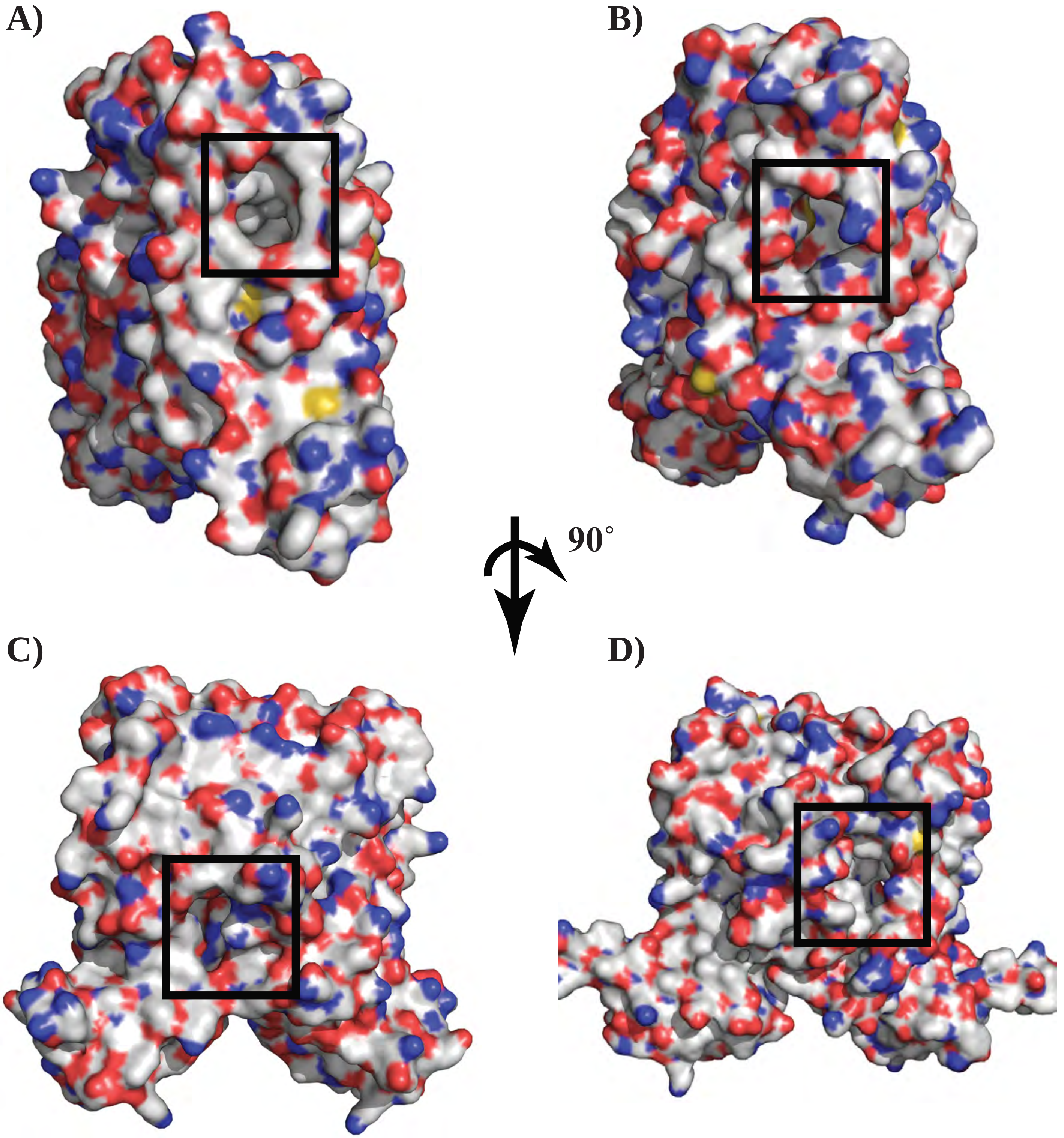

Supplemental Figure S3. Comparison of the likely entry points of germane ligands of the multidrug-binding pockets of the structurally characterized TetR family regulators. (A) Molecular surfaces of the dimers of MtrR, (B) TtgR from Pseudomonas putida (PDB code: 2UXP), (C) QacR from Staphylococcus aureus (PDB code: 1JT6), and (D) CmeR from Campylobacter jejuni (PDB code: 2QCO), as calculated by PyMol (65). The location of the ligand binding pocket is marked by a box. Relative orientation of the structures in the top panels to bottom panels, is shown after a 90˚ rotation around y-axis. The colors blue and red correspond to positive and negative charges, respectively. (White represents neutral charge.) The yellow patches on the structures indicate cysteine residues.

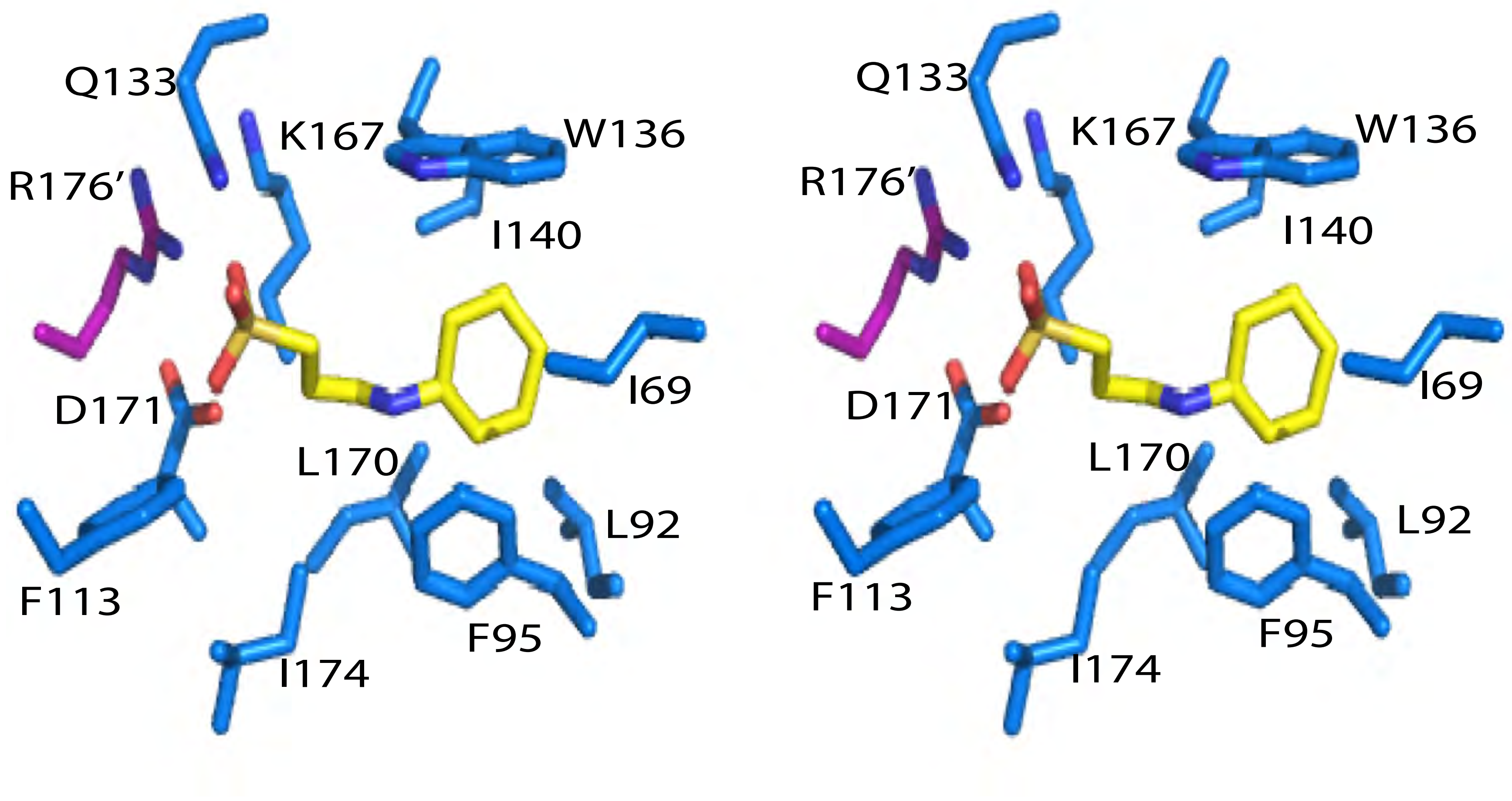

Supplemental Figure S4. Stereo view of possible interactions between CAPS and MtrR. Potential binding residues of MtrR are displayed as sticks in blue and purple corresponding to the two subunits. CAPS is shown in yellow.

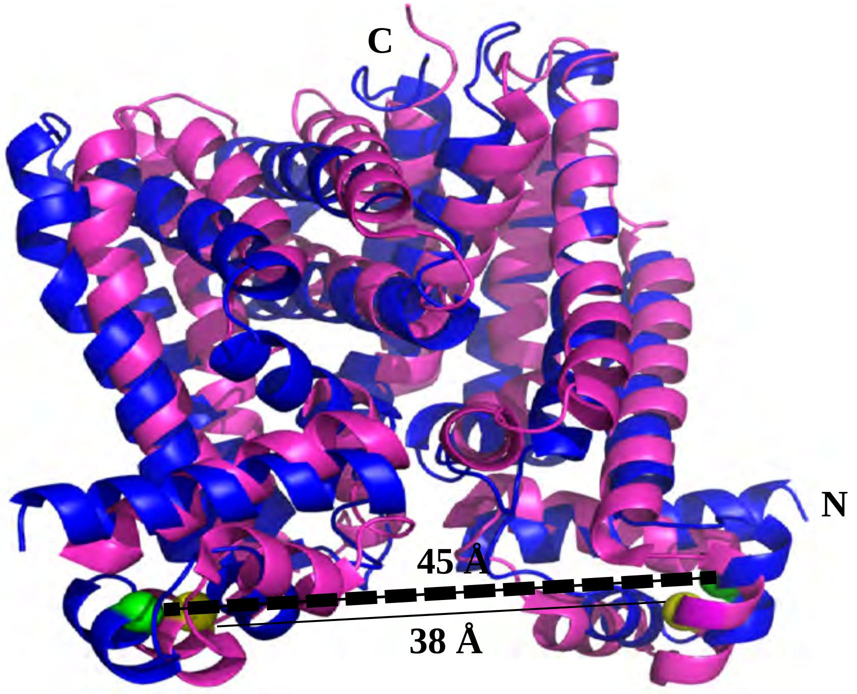

Supplemental Figure S5. Superimposition of the dimers of CAPS-bound MtrR (blue) and DNA-bound QacR (magenta, PDB code: 1JTO) using all corresponding Cα atoms. The midpoints of the recognition helices of MtrR and QacR are shown as spheres. Intersubunit distances (in ängstrom, Å) between the center of the recognition helices of MtrR (dashed lines) and the center of the recognition helices of QacR (solid line) are indicated.

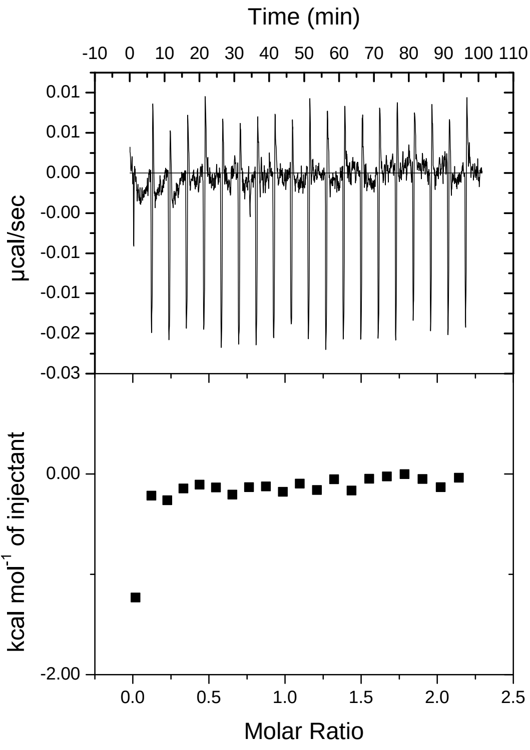

Supplemental Figure S6. Isothermal titration calorimetry thermogram and resulting isotherm for the titration of GCA into MtrR.

A)

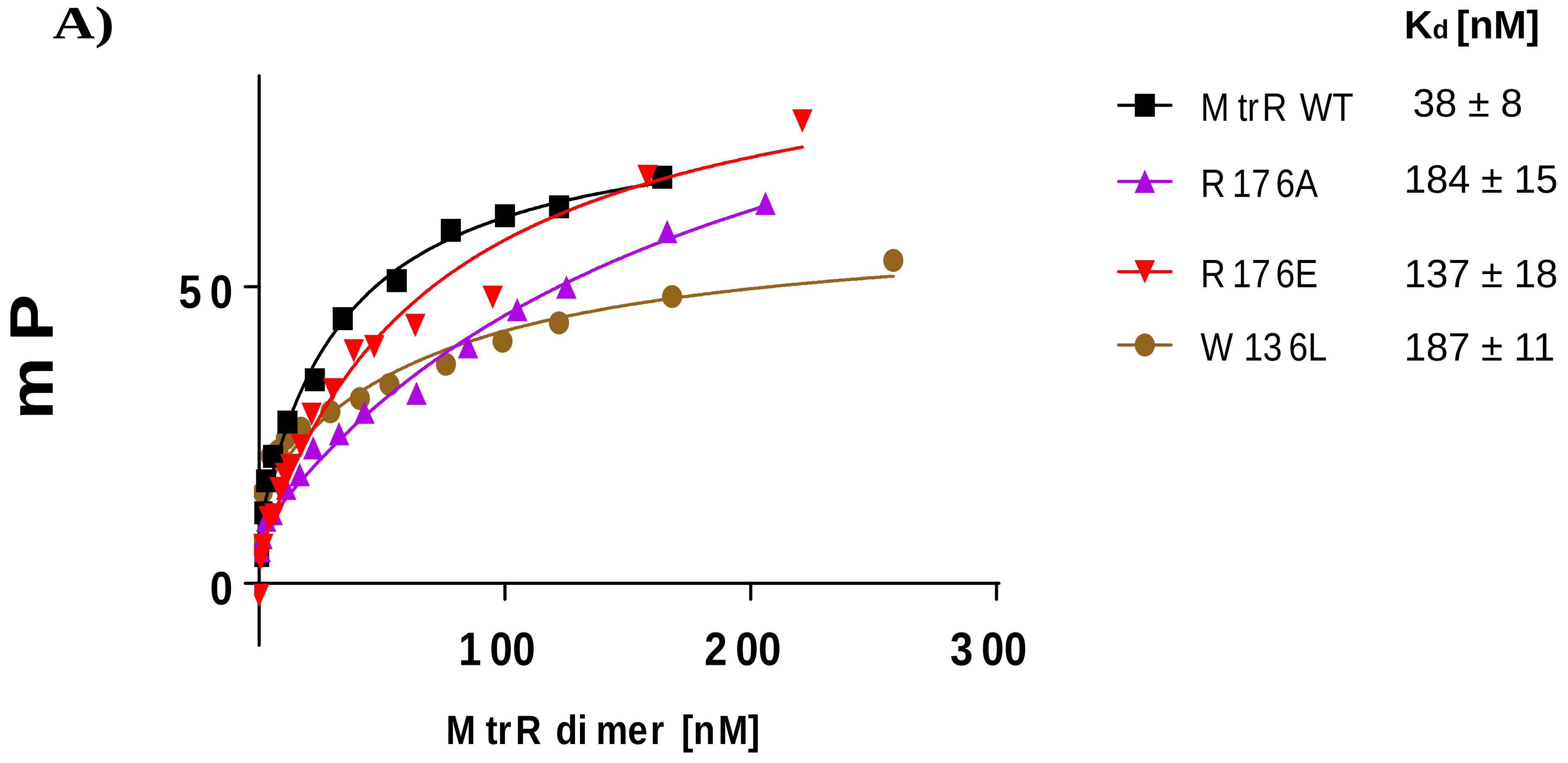

B)

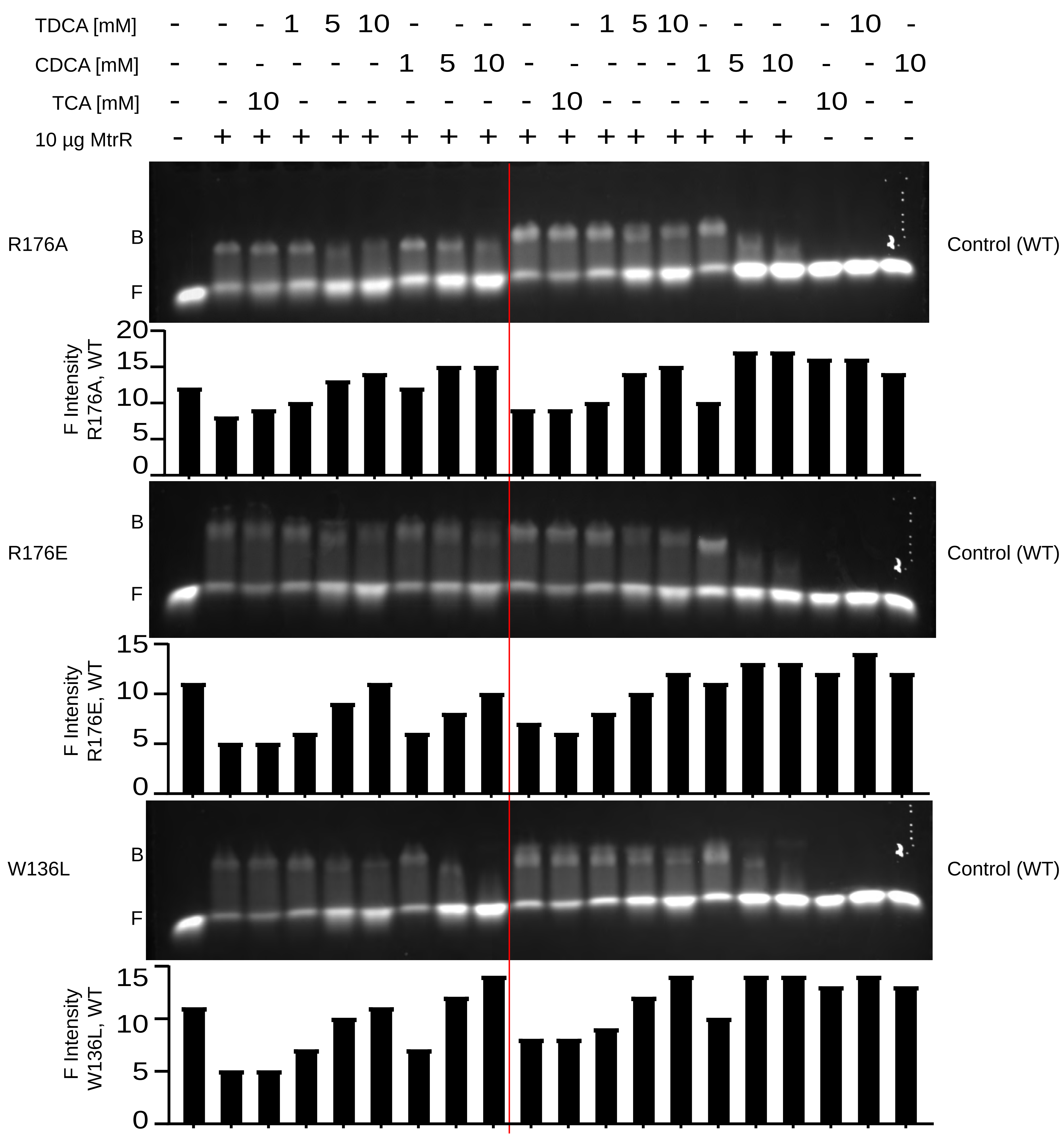

Supplemental Figure S7. DNA-binding and induction of MtrR mutants R176A, R176E, and W136L. (A) Data from fluorescence polarization-based assays showing binding curves and dissociation constants between the mtrCDE operator and MtrR wild-type (WT), R176A, R176E, and W136L. (B) Gel retardation assays to assess the induction capabilities of TDCA, CDCA and TCA (non-inducer control) on R176A, R176E, and W136L MtrR mutant proteins. MtrR WT is juxtaposed as a control. The red line divides the lanes containing one of the single site mutants from those containing MtrR WT. The positions of free probe and MtrR-bound probe are labelled F and B, respectively. Reaction volume was resolved on a 2% agarose gel and analysed by staining with ethidium bromide. The intensity (arbitrary units) of the freely migrating DNA was measured with ImageJ using a fixed number of pixels and is displayed below each corresponding lane in the form of a bar graph.

A)

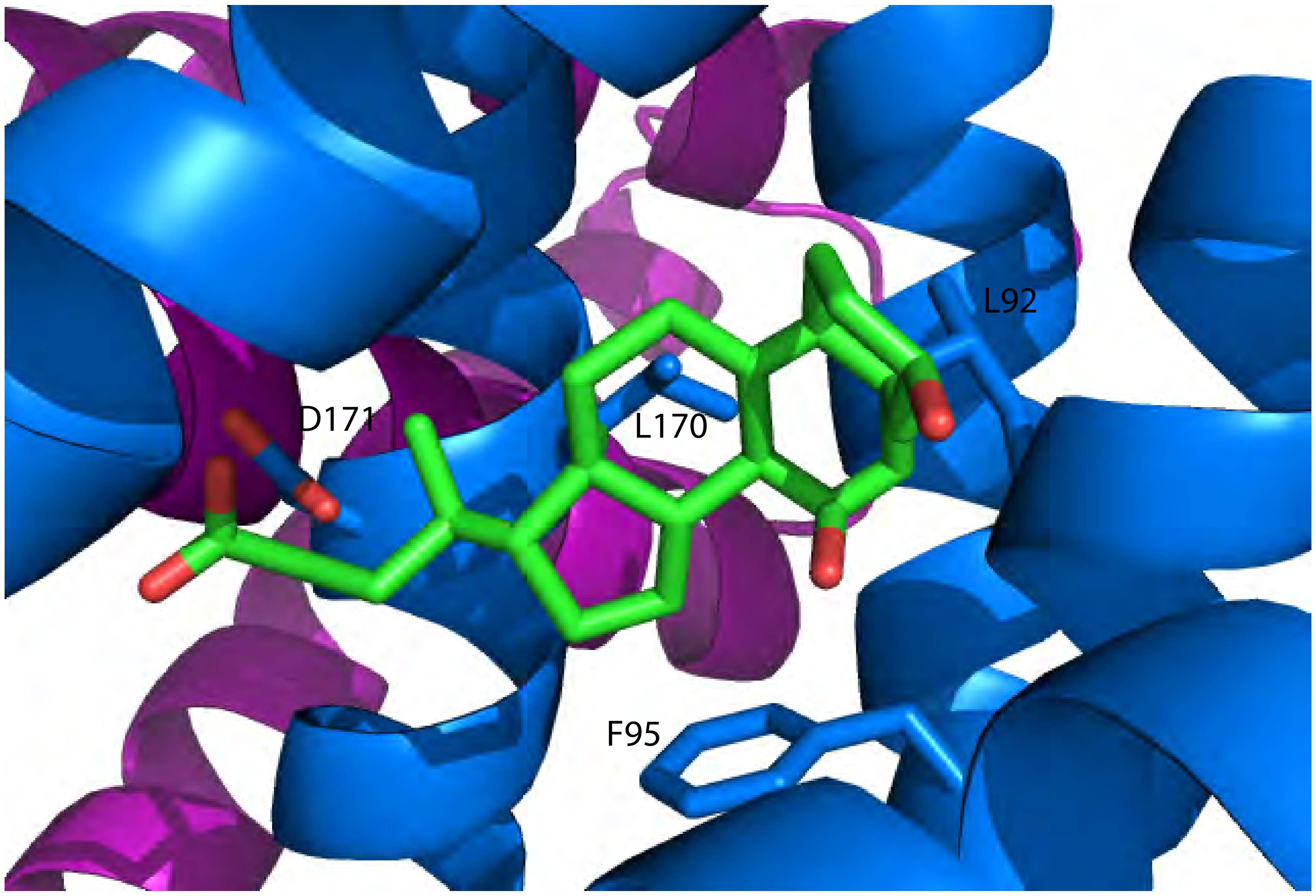

B)

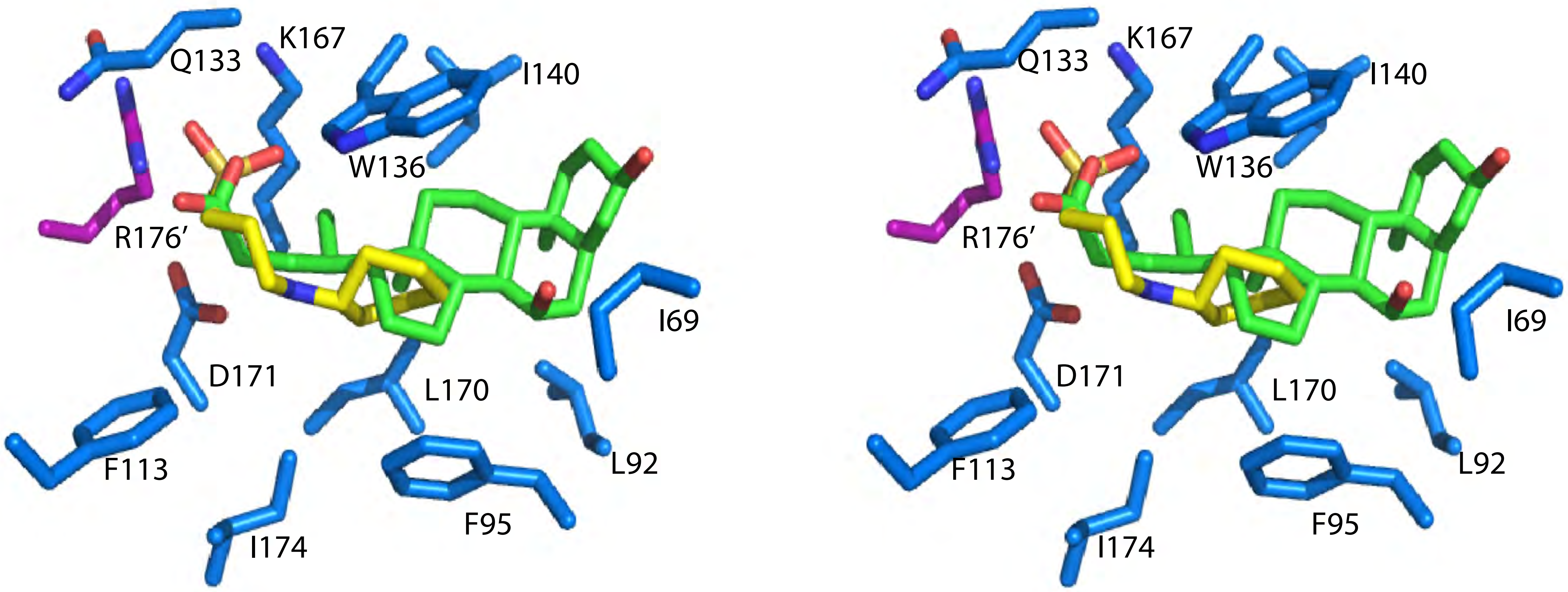

Supplemental Figure S8. Chenodeoxycholate inside the CAPS-binding pocket. CDCA was fit into the binding pocket to minimize steric clashes; the ligand was not energy-minimized. CAPS is shown in yellow and chenodeoxycholate is shown in green; MtrR subunits are shown in purple and blue. (A) A view of the binding pocket that shows the α-helices that form the binding pocket with a few key residues displayed as sticks. (B) A stereo view of the binding pocket with all major residues making up the CAPS-binding pocket displayed as sticks. Overlay of CDCA and CAPS within the pocket.

Supplementary Table S1: Measured parameters from isothermal titration calorimetry experiments. Protein samples including the wild-type and single mutant forms of MtrR are listed in the column on the left and the K_d, stoichiometry, enthalpy and entropy associated with the binding of taurodeoxycholate and chenodeoxycholate are listed in the columns on the right.

	**Taurodeoxycholate**	**Chenodeoxycholate**
**WT**		
K_d [µM]	5.1 ± 1.2	4.4 ± 1.2
N	0.583 ± 0.111	0.444 ± 0.097
ΔH [cal/mol]	-4807 ± 674	-4127 ± 739
ΔS [cal/mol/deg]	8.17 ± 2.68	10.85 ± 3.13
**R176E**		
K_d [µM]	16 ± 3.6	97 ± 64*
N	0.700 ± 0.171	0.521 ± 0.038
ΔH [cal/mol]	-7124 ± 1631	-12979 ± 8228
ΔS [cal/mol/deg]	-1.84 ± 6.07	-23.00 ± 31.48
**W136L**		
K_d [µM]	6.2 ± 1.0	No binding
N	0.440 ± 0.013	
ΔH [cal/mol]	-6458 ± 2969	
ΔS [cal/mol/deg]	2.9 ± 11.0	

*Signal was very weak which could contribute to high error

Supplemental Methods

Protein purification for Isothermal Titration Calorimetry experiments and mutant MtrR proteins

A codon optimized gene of MtrR was synthesized by GenScript and cloned into the pMCSG7 vector in-frame with the N-terminal Hexa-histidine tag and tobacco etch virus (TEV) protease cleavage site. This construct was used as the template for site-directed mutagenesis and to generate wild-type data in supplemental figures 5-6 and Isothermal Titration Calorimetry data. This protein was overexpressed and purified as described in the “Materials and Methods” of the document, except the hexa-histidine tag MtrR was cleaved by TEV protease following NiNTA affinity chromatography. Cleaved MtrR was purified from tagged MtrR and cleaved hexa-histidine tag by Ni-NTA affinity chromatography and then further purified by size exclusion chromatography (S200).

Site-directed mutagenesis

To introduce the mutations W136L, R176A, and R176E, we performed DpnI mediated site-directed mutagenesis. Complementary oligonucleotides containing the desired mutations were obtained from IDT. PCR reactions containing two complementary primers, template DNA, Pfu DNA polymerase buffer, dNTP, and Pfu DNA polymerase were incubated at 95 ºC for 30 seconds followed by 18 cycles of 95 ºC for seconds, 55 ºC for one minute, and 68 ºC for 11 minutes. After cooling the reactions to 4 ºC, 10 units of Dpn1 (NEB) was added to each 50 µl reaction and incubated at 37 ºC for 3 hours. The resulting plasmids were used to transform DH5α (Thermo Fisher) cells for plasmid prep. After checking the sequences to ensure the mutations had been incorporated, these plasmids were overexpressed in Rosetta Gami B(DE3) pLysS cells (Novagen)

and purified following the same protocol as that describing the overexpression and purification of the wild-type construct.
